# Localized Subarachnoid Purulence Mimicking Aneurysmal Subarachnoid Hemorrhage: A Case Report

**DOI:** 10.7759/cureus.98961

**Published:** 2025-12-11

**Authors:** Ken Shinno, Hitoshi Fukuda, Fumihiro Hamada, Masaki Yokodani, Yu Kawanishi, Namito Kida, Naoki Fukui, Masahiro Komori, Yu Arakawa, Mitsuko Iguchi, Yuka Yamagishi, Tetsuya Ueba

**Affiliations:** 1 Department of Neurosurgery, Kochi Medical School Hospital, Nankoku, JPN; 2 Department of Otolaryngology, Kochi Medical School Hospital, Nankoku, JPN; 3 Department of Clinical Infectious Diseases, Kochi Medical School Hospital, Nankoku, JPN; 4 Department of Diagnostic Pathology, Kochi Medical School Hospital, Nankoku, JPN

**Keywords:** aspergillus, endoscopic sinus surgery, intracranial aneurysm, sphenoid sinusitis, subarachnoid hemorrhage, subarachnoid purulence, systemic inflammation, vascular invasiveness

## Abstract

Subarachnoid purulence is generally formed secondary to the rupture of brain abscesses. Early diagnosis of subarachnoid purulence unrelated to brain abscesses is challenging because of the lack of specific neurological or radiological findings. In this article, we report a case of localized subarachnoid purulence mimicking aneurysmal subarachnoid hemorrhage. A 74-year-old woman with diabetes and a history of rhinosinusitis presented with a sudden deterioration of the level of consciousness, tonic posture, and fever. A head computed tomography (CT) scan revealed a high-density area in the left Sylvian fissure. Subsequent CT angiography revealed an ipsilateral posterior communicating artery aneurysm, suggesting a diagnosis of aneurysmal subarachnoid hemorrhage. Craniotomy for surgical clipping of the presumed ruptured aneurysm revealed a subarachnoid purulence rather than a clot. Antimicrobial agents were administered, and endoscopic sinus surgery was performed to control the infection. Histopathological and microbiological examinations of the sphenoid sinusitis and intracranial specimen revealed *Aspergillus*,* Prevotella intermedia*, and *Staphylococcus aureus*. The functional outcome was poor because of delayed cerebral infarction associated with vascular invasion by the subarachnoid purulence.

Localized subarachnoid purulence could be misdiagnosed as aneurysmal subarachnoid hemorrhage, particularly when an incidental aneurysm is detected. Laboratory and physical findings of systemic inflammation, as well as a history of rhinosinusitis, may help physicians add magnetic resonance imaging and lumbar puncture for early and definitive diagnosis of subarachnoid purulence.

## Introduction

Among infectious diseases of the intracranial subarachnoid space, subarachnoid purulence technically refers to deposition or precipitation of purulent matter, while meningitis indicates distribution and dilution of the pathogens in the cerebrospinal fluid. Subarachnoid purulence mostly occurs secondary to the rupture of brain abscesses [1]. However, when subarachnoid purulence occurs unrelated to the brain abscess, definitive diagnosis at the earliest stage is not straightforward because of nonspecific neurological and radiological findings. As the prognosis of subarachnoid purulence is generally poor, early diagnosis and intervention are crucial [2,3]. In this article, we report a case of localized subarachnoid purulence associated with fungal sphenoid sinusitis, which was misdiagnosed as aneurysmal subarachnoid hemorrhage. A localized subarachnoid high-density area and the presence of the adjacent cerebral aneurysm on preoperative imaging led to the initial diagnosis of aneurysmal subarachnoid hemorrhage, but a craniotomy for the presumed culprit intracranial aneurysm revealed subarachnoid purulence. Despite the administration of an antimicrobial agent and endoscopic sinus surgery, the functional outcome was poor due to the vascular invasiveness of the subarachnoid purulence. We discuss the causative pathogens, route of spread, and considerations for differential diagnosis in the acute stage of localized subarachnoid purulence.

## Case presentation

**Figure 1:**
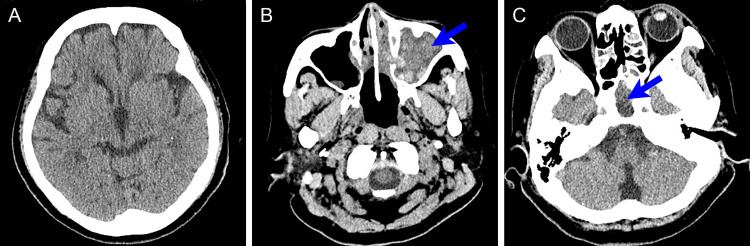
Head CT scan obtained 1 month prior to the ictus when the patient complained of a chronic headache (A) Plain head CT scan at the foramen of Monro level shows no abnormal findings. (B, C) Plain head CT scan at the parasinus level shows maxillary (B) and sphenoid sinusitis (C) (arrows), with mixed high- and low-density areas. No bony destruction is observed. CT: computed tomography

A 74-year-old woman with a history of diabetes mellitus visited the neurosurgical clinic of another hospital with a chronic headache. Head computed tomography (CT) showed no intracranial lesions (Figure 1A) but revealed sphenoid and maxillary sinusitis with mixed high- and low-density areas, without bony destruction (Figures 1B, 1C).

The patient was referred to an otolaryngology clinic for evaluation of the sinusitis but did not follow up. One month later, she was transferred to our stroke center with a sudden-onset severe headache followed by a decreased level of consciousness. Neither vomiting nor neck stiffness was observed. She was comatose with no audible response. She withdrew her bilateral extremities from pain with occasional decorticate posture and upward deviation of the eyeballs. She was febrile at 39.1°C, and her blood pressure, heart rate, and respiratory rate were 151/97 mmHg, 106/min, and 16/min, respectively. Laboratory investigations showed a C-reactive protein level of 29.37 mg/dL, white blood cell count of 16,200/𝜇L, and glucose of 320 mg/dL, which are summarized in Table 1 with other laboratory findings.

**Table 1:** Laboratory findings of the patient

	Value	Reference value (unit)
Hemoglobin	10.3	11.6-14.8 g/dL
White blood cell	16,200	3,300-8,600/𝜇L
Platelet	18.7	15.8-34.8 × 10,000/𝜇L
Glucose	320	73-109 mg/dL
Hemoglobin A1c	7.4	4.9%-6.0%
Creatinine	0.8	0.46-0.79 mg/dL
C-reactive protein	29.37	≤0.14 mg/dL
Serum sodium	134	138-145 mmol/L
Serum potassium	3.7	3.6-4.8 mmol/L
Serum chlorine	103	101-108 mmol/L

**Figure 2:**
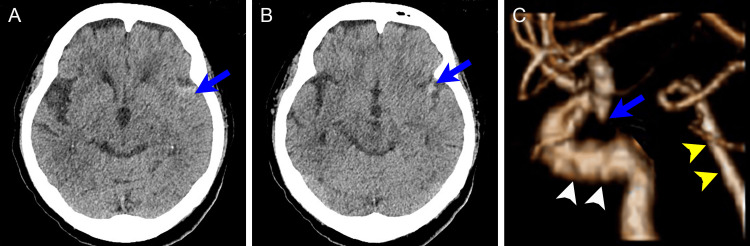
Head CT scan obtained at the symptom onset (A, B) Plain head CT scan shows high-density deposits in the left Sylvian fissure (arrows). Sulcal effacement of the left cerebral hemisphere was observed. (C) A lateral view of the 3D-reconstructed CT angiogram shows a small left posterior communicating artery aneurysm (arrow). White and yellow arrowheads indicate the internal carotid artery and the basilar artery, respectively.

Head CT revealed a high-density deposit in the subarachnoid space of the left Sylvian fissure with no intraparenchymal lesions. Sulcal effacement of the left cerebral hemisphere was observed, indicating the subarachnoid deposit extended to the cortical surface (Figures 2A, 2B). Subsequent CT angiography showed a small left posterior communicating artery aneurysm, and all the cervical and intracranial major arteries were patent (Figure 2C).

She was diagnosed with an acute subarachnoid hemorrhage caused by a ruptured left posterior communicating artery aneurysm. She was immediately intubated and sedated. According to the institutional protocol for aneurysmal subarachnoid hemorrhage, neither head magnetic resonance imaging (MRI) nor lumbar puncture was performed to avoid rebleeding of the aneurysm and delay of neurosurgical intervention. Her severe neurological deficit despite the small amount of subarachnoid blood and high fever was considered to be attributable to postictal status, aspiration pneumonia, or systemic inflammatory response syndrome, associated with acute subarachnoid hemorrhage. She was sent to the operating room for surgical clipping of the ruptured left posterior communicating artery aneurysm to prevent rebleeding. The aneurysm was not considered amenable to endovascular coiling because of its small size. This decision was made solely off of CT angiography, without performing catheter angiography.

**Figure 3:**
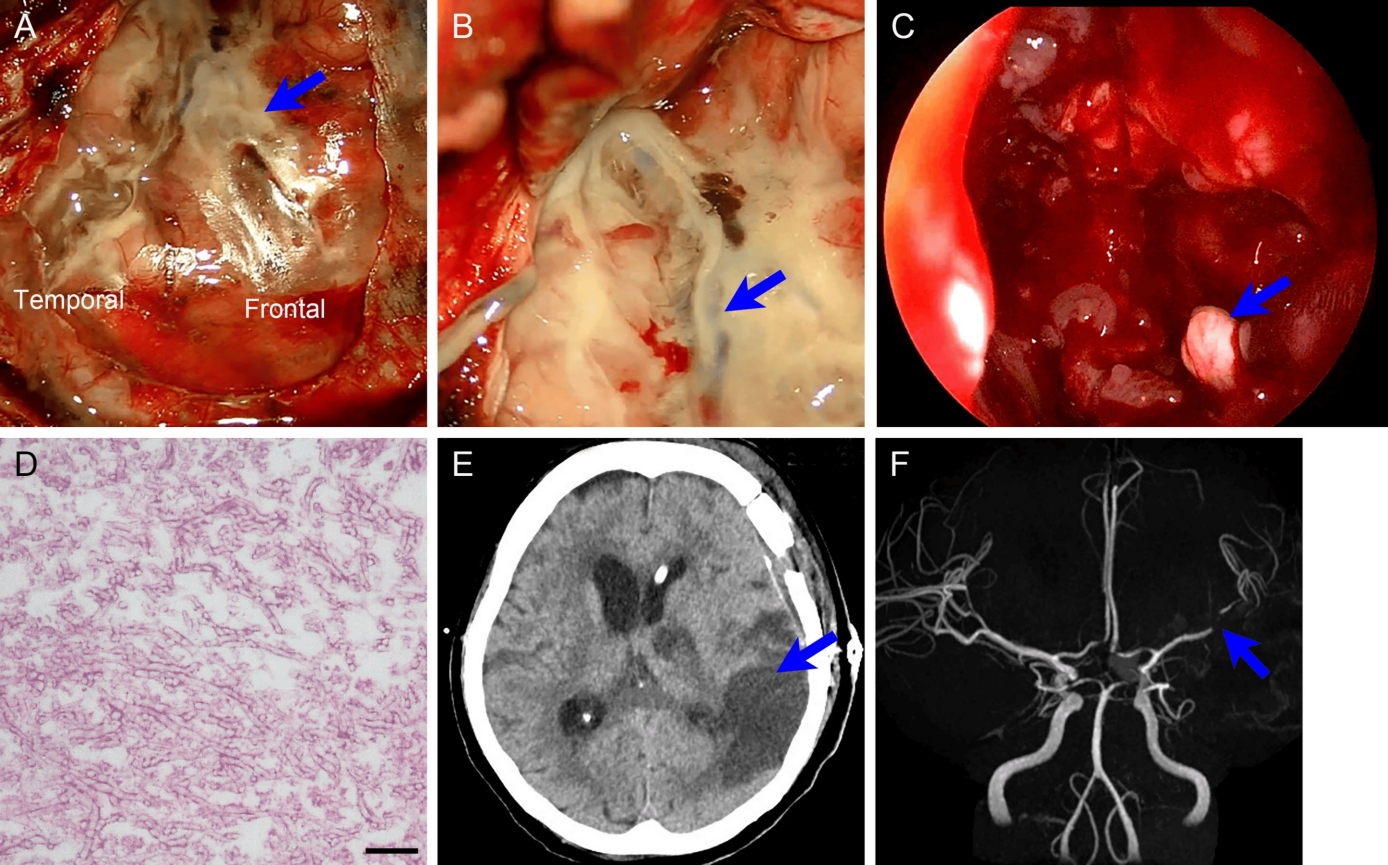
Surgical findings and postoperative course of craniotomy and endoscopic parasinus surgery (A) Dural opening after left frontotemporal craniotomy reveals diffuse yellowish deposits in the left Sylvian fissure and at the adjacent brain surface (arrow). Frontal: left frontal lobe; temporal: left temporal lobe. (B) Microscopic view of the left Sylvian fissure shows collapse of the Sylvian vein (arrow), surrounded by creamy subarachnoid purulence. (C) Endoscopic view of the sphenoid sinus shows a fungal ball (arrow). (D) Higher magnification of the fungus ball with periodic acid-Schiff (PAS) stain shows numerous nonpigmented hyphae, 3-6 𝜇m in diameter, some of which display multiple true septations and characteristic branching at acute angles (45°). Histological examination of the fungal ball with PAS staining reveals mold mycelia with many sharply branched septae, suggestive of *Aspergillus*. Scale bar: 20 𝜇m. (E) Head CT on the fifth day after the onset reveals diffuse ischemic change in the left cerebral hemisphere. (F) Head MR angiogram shows narrowing of the left middle cerebral artery caused by vasospasm (arrow). MR: magnetic resonance; CT: computed tomography

Emergency surgery was performed under general anesthesia. Following a left frontotemporal craniotomy, the dura mater was opened. The subarachnoid space of the left Sylvian fissure was found to be filled with yellowish, sticky deposits, suggesting subarachnoid purulence; no blood clots were observed (Figure 3A). The subarachnoid purulence extended proximally to the anterior-middle fossa base and distally beyond the dural incision along the left Sylvian fissure. The superficial middle cerebral vein had collapsed because of the surrounding subarachnoid purulence (Figure 3B). We collected samples from the subarachnoid purulence, irrigated the subarachnoid space, and completed the surgery. We did not reach the aneurysm because we considered it unruptured.

The following day, her history of sphenoid and maxillary sinusitis was reported by another hospital, which was then regarded as the cause of the subarachnoid purulence. Retrospective observation of the head CT scan at onset revealed no change in maxillary and sphenoid sinusitis. Endoscopic paranasal sinus surgery was performed for the sphenoid and maxillary sinusitis. Her left middle meatus was filled with an abscess, and all the paranasal sinuses were opened and irrigated. No bony defects were observed. A large abscess, including a whitish mass suggestive of a fungal ball, was found in the sphenoid sinus (Figure 3C). *Prevotella intermedia*, a member of the normal oral bacterial flora, and *Staphylococcus aureus* were detected in the culture of the subarachnoid purulence and the parasinus abscess, respectively. Histological examination of the mass in the sphenoid sinus revealed *Aspergillus* (Figure 3D). Consequently, she was diagnosed with a subarachnoid purulence caused by multiple bacteria transferred from the sphenoid sinus with the aid of invasive primary *Aspergillus* sinusitis. She was treated with six-week antimicrobial agents, starting with meropenem, vancomycin, and liposomal amphotericin B, with subsequent de-escalation to ceftriaxone, metronidazole, and voriconazole. A head CT scan obtained on the fifth day after the onset revealed diffuse ischemic changes in the left cerebral hemisphere, which were caused by narrowing of the left middle cerebral artery, as depicted by magnetic resonance angiography (Figures 3E, 3F). She remained unconscious and bedridden during this period and was transferred to a long-term nursing facility on the 45th day after onset.

## Discussion

Most subarachnoid purulence is caused by rupture of previously formed brain abscesses [1]. However, when unrelated to the brain abscess, early diagnosis is challenging due to the lack of specific radiological and neurological findings. Also, the prognosis of subarachnoid purulence is generally poor due to its vascular invasiveness, and surgical intervention including drainage is ineffective or risky due to its diffuse distribution, unlike walled-off brain, epidural, or subdural abscesses [2-5]. In the present case, the pathogens were considered to have been transferred intracranially through the transvenous route from acute deterioration of chronic sphenoid sinusitis. Intracranial extension of pathogens from rhinosinusitis, including epidural, subdural, and brain abscesses, meningitis, and cavernous sinus thrombosis, is referred to as rhinogenic intracranial complications [3]. Rhinogenic intracranial complications occur in approximately 3% of hospitalized patients with rhinosinusitis and are associated with high morbidity and mortality, especially when diagnosis and treatment are delayed [2,3]. Spontaneous rhinogenic intracranial complications in patients with no history of traumatic injury or sinus surgery are generally attributed to frontal or sphenoid sinusitis [4,6]. Frontal sinusitis is predisposed to cause epidural, subdural, or brain abscesses by direct invasion of the pathogens through the thin cribriform plate, whereas sphenoid sinusitis causes meningitis or cavernous sinus thrombosis by hematogenous transfer through the emissary veins [7,8]. There are epidemiological differences in this regard. Intracranial complications owing to frontal sinusitis are predominantly observed in young males, whereas those owing to sphenoid sinusitis are more likely to occur in the elderly [5].

Most intracranial transfers of the pathogens from the sphenoid sinusitis only cause meningitis or cavernous sinus thrombosis [7,8]. Thus, the precise mechanism of how co-infection of fungus and bacteria contributed to subarachnoid invasion of the purulence is of particular concern. Among fungal sinusitis, which is generally less invasive and has a favorable prognosis, *Aspergillus *sinusitis is aggressive and prone to extend into the intracranial or intraorbital space [9]. We assume that acute deterioration of *Aspergillus *sphenoid sinusitis facilitated the development and mediated aggressive subarachnoid progression of the aerobic and anaerobic bacteria through the sphenoparietal sinus and superficial middle cerebral vein [4]. Direct transfer, which generally causes epidural, subdural, and frontal lobe abscesses by direct contact, was less likely because of the lack of bony destruction at the sphenoid sinus wall. Once subarachnoid purulence occurs, an intense inflammatory vasculitis results in cerebral infarction and poor prognosis. In addition, an immunocompromised status associated with older age and a history of diabetes mellitus may have contributed to the intracranial extension. Invasive rhinosinusitis caused by *Aspergillus* is characterized by a mosaic appearance of high- and low-density areas on CT scans [10]. Thus, immediate intervention may be recommended for sphenoid sinusitis with such CT findings to prevent intracranial invasion [11].

In the present case, rhinogenic intracranial complications revealing a high-density area in the subarachnoid space were not diagnosed until a craniotomy was performed for the aneurysm. Previous literature described high-density CT signals of localized subarachnoid purulence or meningitis [12-14], presumably due to high protein concentration of the purulent exudate. When a head CT scan of a patient with acute-onset deterioration of the level of consciousness shows a high-density area exclusively in the subarachnoid space, the most likely diagnosis is aneurysmal subarachnoid hemorrhage. This diagnosis was further supported by the subsequent CT angiography, which revealed a posterior communicating artery aneurysm on the same side as the subarachnoid lesion. Since rebleeding of a ruptured aneurysm is one of the determinants of poor outcomes in aneurysmal subarachnoid hemorrhage, early intervention by endovascular embolization or surgical clipping is recommended [15]. In this situation, diagnosing localized subarachnoid purulence, which has a low prior probability and lacks specific clinical or radiological findings, is challenging. However, the present case gives several important insights into differential diagnosis between subarachnoid purulence and aneurysmal subarachnoid hemorrhage. First, laboratory and physical findings of systemic inflammation, including elevated C-reactive protein and white blood cell count, fever, and marked decrease in the level of consciousness, are rather suggestive of severe infection. These findings are rarely observed in aneurysmal subarachnoid hemorrhage with localized clots, although possible when accompanying epileptic seizures, aspiration pneumonia, or systemic inflammatory response syndrome [16]. In addition, a small ipsilateral aneurysm has a lower possibility of rupture. With such atypical findings, the institutional protocol for aneurysmal subarachnoid hemorrhage may be flexibly applied so that head MRI may provide additional information, and, more importantly, lumbar puncture may reveal bacteria rather than xanthochromia for early definitive diagnosis of subarachnoid purulence [13]. In the present case, surgical clipping was performed for a small aneurysm less amenable to endovascular embolization, thus confirming subarachnoid purulence through direct observation via craniotomy [17]. An antimicrobial agent was administered and endoscopic sinus surgery was performed immediately after craniotomy to control the infection. Nevertheless, diffuse cerebral infarction, presumably caused by vascular invasion of the pathogens into cerebral arteries, led to poor functional outcomes for the patient.

## Conclusions

Here, we reported a case of localized subarachnoid purulence mimicking aneurysmal subarachnoid hemorrhage. *Aspergillus* sphenoid sinusitis, which is invasive, can cause localized subarachnoid purulence via the transvenous route through the cavernous sinus and superficial middle cerebral vein in older patients with diabetes mellitus. Once this occurs, the functional outcomes of subarachnoid purulence are generally poor owing to the vascular invasiveness of the cerebral arteries. In the present case, a localized subarachnoid high-density area on CT scan, a concurrent incidental intracranial aneurysm, and a lack of information about the history of sinusitis caused our obsession with aneurysmal subarachnoid hemorrhage. However, because laboratory and physical findings of systemic inflammation, as well as configuration of the concurrent aneurysm, were atypical, the addition of MRI and lumbar puncture would have led to early definitive diagnosis of subarachnoid purulence, which are lessons we can learn from this case report.
